# Intra-Abdominal Spilled Gallstones Mimicking Malignancy: A Case Report and a Literature Review

**DOI:** 10.7759/cureus.32376

**Published:** 2022-12-10

**Authors:** Maha S Almslam, Abdulmalik I Alshehri, Abdullah A Alshehri, Musthafa C Peedikayil, Khalid M Alkahtani

**Affiliations:** 1 Department of Medicine, Alfaisal University College of Medicine, Riyadh, SAU; 2 Department of Medicine, King Saud Bin Abdulaziz University for Health Sciences College of Medicine, Riyadh, SAU; 3 Department of Medicine, King Faisal Specialist Hospital and Research Centre, Riyadh, SAU

**Keywords:** peritoneal gallstone, intra-abdominal mass, lap cholecystectomy, spilt gallstones, retained gallstones

## Abstract

The incidence of gallstone spillage and gallbladder perforation has increased as a result of the rising use of laparoscopic cholecystectomy. The presence of gallstones in the abdomen may lead to adhesions, inflammation, infection, and obstruction of biliary ducts. Since different etiologies can occur with spillage of gallstones, variation in presentation is expected. We report a case of a laparoscopic cholecystectomy complication after four years of surgery. The patient’s clinical presentation mimicked malignancy.

## Introduction

Cholecystitis is an inflammation of the gallbladder, often from obstruction of the cystic duct, and usually presents with right upper quadrant pain, and is often treated surgically [1]. The surgical approach is usually done laparoscopically rather than open surgery due to its minimal invasiveness, reduced hospitalization, and decrease in morbidity [2]. However, dropped stones (6-30%) and perforation of the gallbladder (10-40%) are potential complications of this procedure [3]. A study showed that 12% of patients were asymptomatic and diagnosed incidentally with gallstone spillage [4]. Internationally, complications secondary to unretrieved spilled gallstones were approximately between 2.3% and 7% [5]. A variety of complications are caused by unretrieved gallstones, varying from simple infection to more severe obstruction [6]. Abscesses made up 56.5% of all cases and were the most frequent sequelae, with intrabdominal abscesses accounting for 36.5%, abdominal wall abscesses at 10.6%, and retroperitoneal abscesses at 9.4% [4]. Patients usually present after months to years secondary to laparoscopic cholecystectomy with nonspecific signs and symptoms [7]. Despite that, cases of dropped stones are usually clinically asymptomatic [8,9]. Following a laparoscopic cholecystectomy, some prominent serious complications of stone spilling might develop. These include fever, broncholithiasis, intestinal obstruction, gallstone granuloma, ileus, abscesses in the liver, and paracolic area, which may present with similar signs and symptoms to malignancy [3,10].

This case report aims to highlight an atypical presentation of a laparoscopic cholecystectomy complication four years (2019) after the procedure was performed and presented with warning signs of malignancy. Physicians should have a high index of suspicion to diagnose the unusual presentations of spillage gallstones to provide the most optimal care without any delay to patients.

## Case presentation

A 34-year-old male who had undergone laparoscopic cholecystectomy four years ago presented with abdominal pain in the right hypochondriac area. Significant weight loss, night sweats, and radiological features raised the possibility of malignancy, and he was referred to our center for further evaluation.

The patient is a known case of bronchial asthma presented after being referred in November 2021 from a private hospital for suspected gastrointestinal malignancy admitted under medical oncology for further evaluation of the one-year history of right upper quadrant pain, night sweats, and weight loss. Biopsy results were suggestive of an inflammatory myofibroblastic tumor for further characterization and management.

Because of the complexity of this patient’s presentation, further detailed history was obtained in our clinic; abdominal pain was located in the right upper quadrant radiating to the back for the past 18 months, worsening with fatty food and coffee consumption. The pain was relieved by medications. Further inquiry regarding patient status before the onset of pain, as the presentation was vague, showed that symptoms had started after laparoscopic cholecystectomy. The patient also reported weight loss of 21 kg since 2019 after restricting oral intake due to pain in addition to bariatric surgery which the patient had undergone before cholecystectomy. The patient complained of drenching night sweats. He reported a history of contact with domestic cats and denied any nausea, vomiting, change in bowel habits, skin rashes, visual symptoms, joint pains, travel history, or sick contact.

The patient underwent imaging for this issue in another private hospital where he was treated for *Pseudomonas* abscesses and infection with intravenous antibiotics. The patient received multiple courses of antibiotics which relieved his pain for a few weeks and then relapsed. The patient was on Symbicort and as needed Ventolin. 

The patient's vital signs on admission revealed a temperature of 36.7°C, a heart rate of 66 beats per minute, a respiratory rate of 20 breaths per minute, oxygen saturation of 98% on room air, and blood pressure of 110/65. During the physical examination, the patient's abdomen was soft and lax without tenderness, and organomegaly or masses were noted on palpation of the abdomen. Normal bowel sounds were heard on auscultation.

Additionally, a laboratory workup was done; a complete blood count was obtained, which showed hemoglobin subunit beta (Hgb) 12.3 g/dL and mild leukocytosis (11.8 × 10^9^/L), and liver function test and renal function test were within normal limits (including albumin, alanine transaminase, aspartate aminotransferase, alkaline phosphatase, creatinine, and electrolyte). Peripheral blood smear showed borderline normocytic normochromic anemia. All blood cultures and tumor markers (cancer antigen 19-9, alpha-fetoprotein tumor, and carcinoembryonic antigen) were negative.

**Figure 1:**
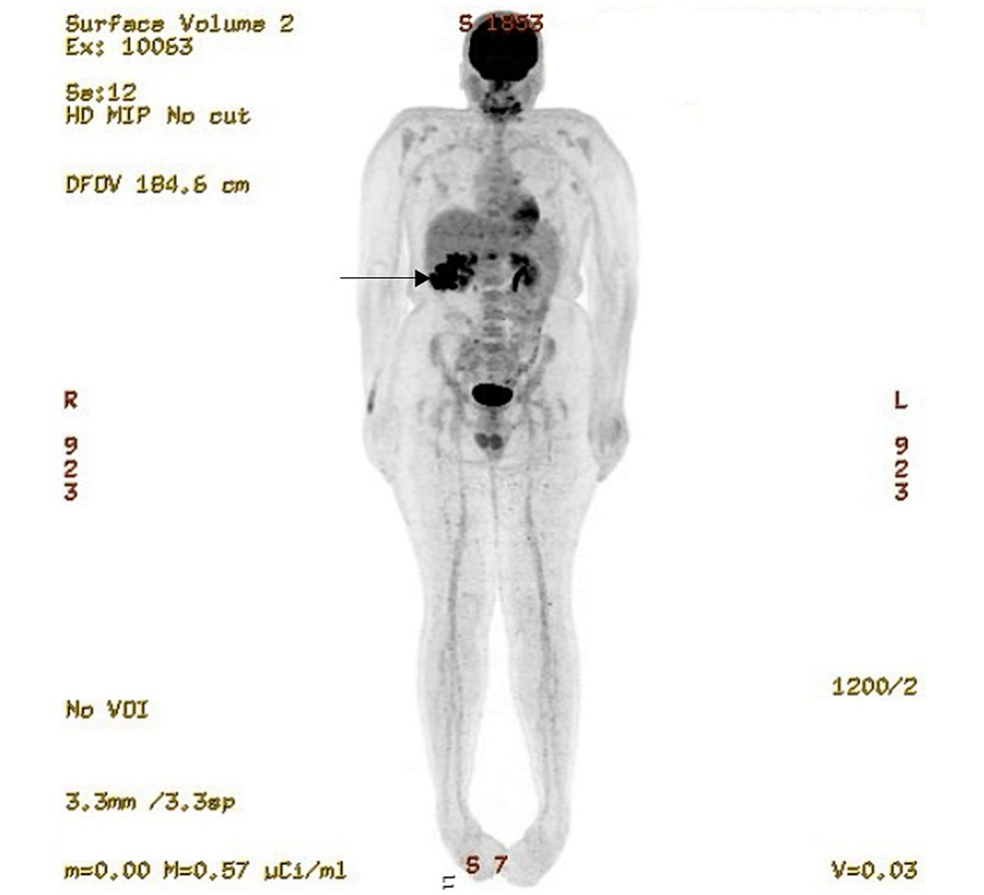
PET CT The image shows marked FDG-avid right abdominal upper quadrant perihepatic/perirenal soft tissue lesions. PET CT, positron emission tomography computed tomography; FDG, F-fluorodeoxyglucose.

**Figure 2:**
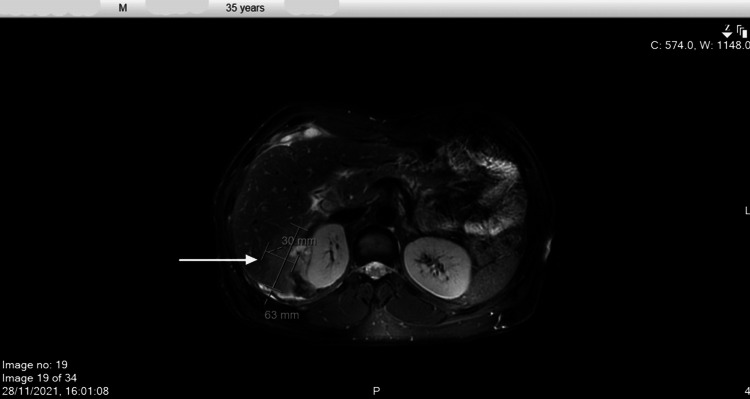
MRI abdomen/pelvic (axial plane) The image shows multifocal infiltrative lesions centered in the hepatorenal space invading the adjacent liver, right kidney, and abdominal wall. MRI, magnetic resonance imaging.

**Figure 3:**
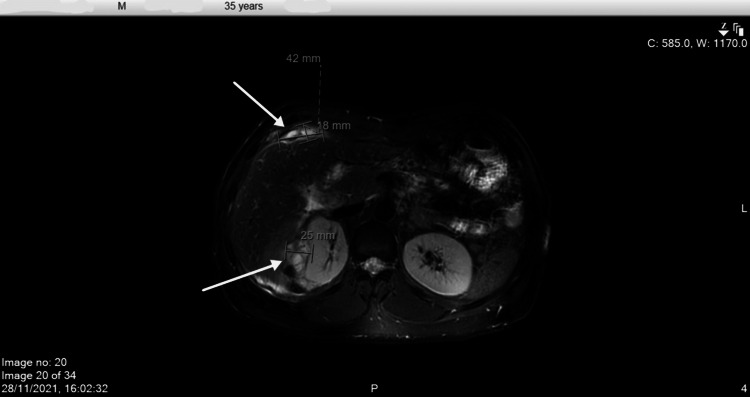
MRI abdomen/pelvic (axial plane) The image shows additional similar perihepatic nodules overlying the left hepatic lobe anteriorly and posteriorly. MRI, magnetic resonance imaging.

Whole-body positron emission tomography-computed tomography (PET-CT) was obtained, which showed noticeably increased 18F-fluorodeoxyglucose (FDG) activity corresponding to the perihepatic/hepatic soft tissue deposits with no definite additional intrahepatic focal FDG-avid lesions (Figure 1). Magnetic resonance imaging (MRI) abdomen was done, which showed multifocal infiltrative lesions centered in the hepatorenal space invading the adjacent liver, right kidney, and abdominal wall, and also similar perihepatic nodules overlying the left hepatic lobe anteriorly and posteriorly (Figures 2, 3). A liver biopsy was done with histopathology showing suspected granulomatous inflammation or pseudotumor.

The patient underwent robotic exploration to excise the inflammatory mass in the hepatorenal space; the frozen section result was negative for malignancy and showed granulomatous inflammation only. During surgery, dense adhesions were encountered where the liver adheres to small bowels and lateral abdominal wall requiring adhesiolysis. The dissected hepatorenal mass revealed thick-walled multiple cystic lesions with caseating cheesy material and pus discharge, and multiple small stones were also found. Finally, fastidious irrigation and drainage was done, and the wall of the cystic lesion was excised in multiple pieces. Regarding medication, the patient was on meropenem, vancomycin, fluconazole, and prophylaxis enoxaparin.

The patient was discharged on the second day after surgery. During the three-month follow-up visit, the patient was doing well and underwent a targeted abdominal ultrasound, which was normal without any visible intra-abdominal collection or mass.

## Discussion

This case shows a common condition with an unusual presentation that causes a delay in diagnosis. Based on this patient’s presentation, chronic pain in the right upper quadrant, weight loss history, and night sweat with the presence of a mass on radiological imaging suggestive of malignancy, the accurate diagnosis was masked. Atypical presentations of gallstone spillage during laparoscopic cholecystectomy are not uncommon; therefore it is important that clinicians must take this into account when establishing differential diagnoses. However, laparoscopic cholecystectomy is considered the "gold standard" for the surgical treatment of symptomatic gallstones. This surgical procedure is correlated with lower perioperative morbidity and mortality and shorter hospital stays [11]. Despite its beneficial advantages, there are still some expected complications that are rather different from open cholecystectomy, like in our case, i.e. the spillage of stones intraoperatively [12].

In contrast to laparoscopic cholecystectomy, during open cholecystectomy, the surgeon is able to retrieve the gallstone by aspiration and irrigation [13]. One study showed that intraoperative spillage of gallstones and bile into the abdominal cavity occurred in 306 (29%) patients out of 1059 [14]. There are some risk factors that are more commonly associated with gallstone spillage, such as advanced age, obesity, and male gender [15]. The gallstone mass was visible in a CT scan of the patient. This particular complication is frequently missed and might present several years later [12]. In another study, only two of the 22 asymptomatic patients had identifiable retained gallstones seen by CT scan in the abdominal cavity [16]. Foreign body granulomas are formed due to the presence of gallstones that cannot be phagocytosed by a single macrophage, and it has been documented in other clinical studies [17]. There have been reports of successful percutaneous removal of retained gallstones, but there are limitations on the size and location of the gallstones [18].

The body treats unremoved gallstones as a foreign body. If the stones were sterile, the inflammation process may lead to a granuloma, and if contaminated, it may cause the formation of an abscess [19]. In another study by Ologun et al. [20], a similar case was presented after four years of laparoscopic cholecystectomy, with epigastric pain for three months, in which labs and physical examination were inconclusive. Subsequently, CT was ordered, which showed a dropped gallstone in the abdomen with a densely calcified lesion inside the omentum with no inflammation around it [20]. Usually, in the case of spilled gallstones, the presentation is vague, and labs are usually within normal limits, with only a positive history of laparoscopic cholecystectomy; however, in such cases, CT and US (ultrasound) imaging are the most useful tools to diagnose gallstones spillage [7]. One of the methods for early detection of laparoscopic cholecystectomy complications is taking detailed surgical history and raising the suspicion of gallstone droppage even months to years after cholecystectomy, which may help to decrease the potential risk of complications of this condition.

## Conclusions

One of the common complications of laparoscopic cholecystectomy is iatrogenic perforation of the gallbladder with spilled gallstones. As the presentation of retained stones during cholecystectomy varies, it is crucial to ask for a detailed history upon the first encounter with the patient. This would aid in providing excellent health care and avoid unnecessary investigations. At that point, our patient would not have suffered from this agony for 18 months if a proper history had been taken when he first presented. Thus, we are highlighting the importance of reporting unusual presentations of similar conditions.
